# Changes in Glycated Human Serum Albumin Binding Affinity for Losartan in the Presence of Fatty Acids In Vitro Spectroscopic Analysis

**DOI:** 10.3390/molecules27020401

**Published:** 2022-01-08

**Authors:** Agnieszka Szkudlarek, Jadwiga Pożycka, Karolina Kulig, Aleksandra Owczarzy, Wojciech Rogóż, Małgorzata Maciążek-Jurczyk

**Affiliations:** Department of Physical Pharmacy, Faculty of Pharmaceutical Sciences in Sosnowiec, Medical University of Silesia in Katowice, 40-055 Katowice, Poland; jpozycka@sum.edu.pl (J.P.); kkulig@sum.edu.pl (K.K.); aowczarzy@sum.edu.pl (A.O.); wrogoz@sum.edu.pl (W.R.); mmaciazek@sum.edu.pl (M.M.-J.)

**Keywords:** spectroscopic methods, glycated human serum albumin, saturated and unsaturated fatty acids, losartan

## Abstract

Conformational changes in human serum albumin due to numerous modifications that affect its stability and biological activity should be constantly monitored, especially in elderly patients and those suffering from chronic diseases (which include diabetes, obesity, and hypertension). The main goal of this study was to evaluate the effect of a mixture of fatty acids (FA) on the affinity of losartan (LOS, an angiotensin II receptor (AT_1_) blocker used in hypertension, a first-line treatment with coexisting diabetes) for glycated albumin—simulating the state of diabetes in the body. Individual fatty acid mixtures corresponded to the FA content in the physiological state and in various clinical states proceeding with increased concentrations of saturated (FA_S_) and unsaturated (FA_US_) acids. Based on fluorescence studies, we conclude that LOS interacts with glycated human serum albumin (af)gHSA in the absence and in the presence of fatty acids ((af)gHSA_phys_, (af)gHSA_4S_, (af)gHSA_8S_, (af)gHSA_4US_, and (af)gHSA_8US_) and quenches the albumin fluorescence intensity via a static quenching mechanism. LOS not only binds to its specific binding sites in albumins but also non-specifically interacts with the hydrophobic fragments of its surface. Incorrect contents of fatty acids in the body affect the drug pharmacokinetics. A higher concentration of both FA_S_ and FA_US_ acids in glycated albumin reduces the stability of the complex formed with losartan. The systematic study of FA and albumin interactions using an experimental model mimicking pathological conditions in the body may result in new tools for personalized pharmacotherapy.

## 1. Introduction

Human serum albumin (HSA), being the main protein in plasma, is essential in many processes taking part in the body. HSA performs key functions in maintaining homeostasis in the body, e.g., HSA controls the plasma oncotic pressure, modulates the fluid distribution between the body compartments, displays antioxidant and enzymatic properties, and inactivates toxic compounds [1,2,3,4]. HSA has the ability to transport many biologically active compounds through binding endo- and the exogenous compounds (e.g., fatty acids, metal ions, drugs, hormones, vitamins, toxins, and metabolites) [1,4].

One of the processes causing the loss of albumins original properties is the increased glycation in a state of hyperglycemia. Heterogeneous, stable compounds formed at the end of this process—Advanced Glycation End-Products (AGEs)—play a significant role in the development of chronic micro- and macroangiopathic diabetic complications as well as degenerative processes related to age [5,6].

Fatty Acids (FAs) perform many important function in living organisms, e.g., they are used as energy substrates in the β-oxidation process; as a building material for phospholipids, which are, in turn, used to create biological membranes; and they are precursors of important biological mediators, such as prostaglandins, leukotrienes, and thromboxanes [4,7]. 

FAs are also involved in intracellular transmission and take part in post-transcriptional modification processes [7]. As components of complex lipids, FAs play an important role in the electric and thermal isolation of the body, as well as provide it with mechanical protection. Due to the fatty acid low solubility in the blood plasma, albumin is the main transporter of FA [8]. The albumin-FA complex is in equilibrium with a very small fraction of unresolved FA dissolved in the plasma (less than 0.01% of the total pool).

**Figure 1:**
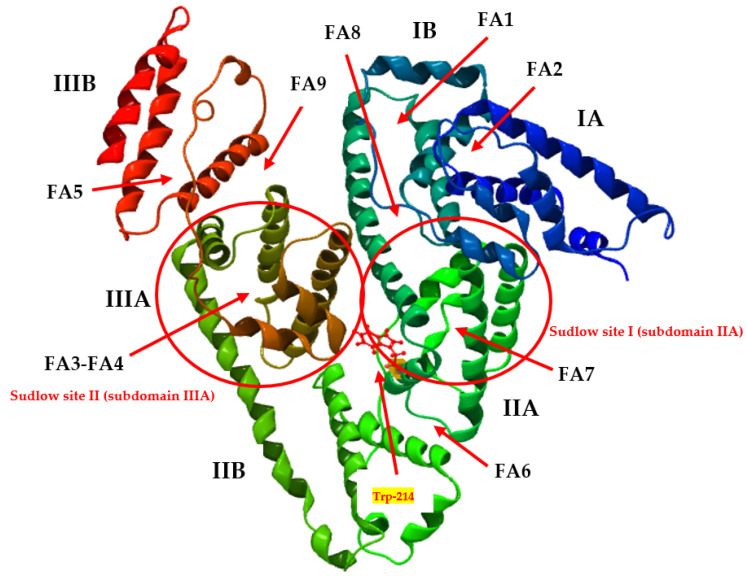
Human serum albumin (HSA) drug binding sites (Sudlow site I and II) with the location of main fatty acids in the HSA molecule (FA1–FA9) and the marked Trp-214 residue. Molecular graphic image based on [11] was produced using the CLC Drug Discovery Workbench version 1.0.2. (CLC Bio, a QIAGEN Company: Aarhus, Denmark) [license number: CLC-LICENSE-51JT8-DXYBY-2A3EW-ED80P-DGW80] (PDB ID: 4K2C).

Apart from the two main versatile ligand binding sites with a high affinity for diverse molecules referred to as ‘Sudlow’s sites’—site I (located in subdomain IIIA) and site II (in subdomain IIA) [9]—there are nine fatty acid binding sites in the HSA molecule (i.e., FA1–FA9), which are arranged in an asymmetrical manner and include all six subdomains [10] (Figure 1).

On one side, the non-polar bonds with fatty acids protect the tertiary structure of albumin against denaturation with guanidine hydrochloride, urea, and temperature. On the other side, there is a conformational change in the macromolecule and an increase in Cys-34 reactivity as a result of the exposure of the sulfhydryl residue [12]. Structural changes in Sudlow’s site I concern the reorganization of hydrogen bonds between amino acid residues Tyr-150, Glu-153, Gln-196, His-242, Arg-257, and His-288, which leads to an increase in the area of the binding pocket and a polarity disorder. 

In Sudlow’s site II, there is a change in the conformation of the Leu-387 and Leu-453, and the breaking of Arg-348 and Glu-450 bonds. This allows the fatty acids, bound at FA3, to gain access to the polar region around FA4 [12]. Unmodified human serum albumin binds anions, when in combination with fatty acids, indicates an increased affinity for substances in the form of cations [13].

Many experiments have shown that the presence of fatty acids can have a significant impact on the process of drugs binding by albumin, particularly drugs with a high affinity for macromolecules. Fatty acids can compete for the HSA molecule binding sites or cooperate with drugs, wherein the FA affinity for albumin decreases with every filled macromolecule binding site [14,15]. 

It is important as ligands bound to one binding site can change the structure or number of other binding sites in the albumin molecule [12]. Due to the diversity present in the results concerning change in pharmacological action through research revolving around in vitro and in vivo studies of exogenous ligand interactions, the binding mechanisms of drugs in the presence of fatty acids with a transport protein requires thorough study.

**Figure 2:**
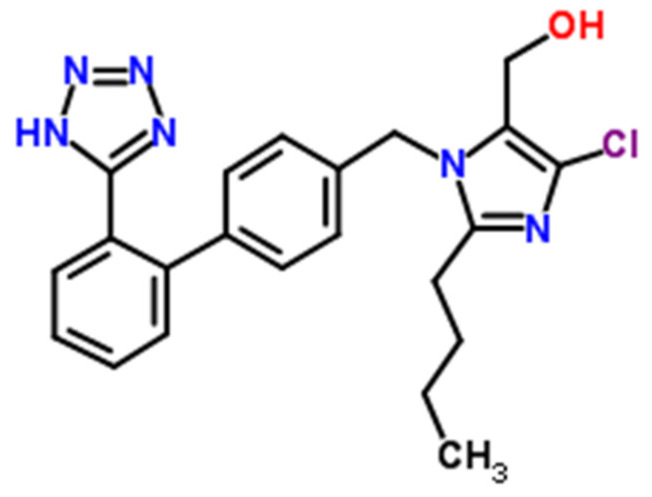
The chemical structure of losartan ((2-Butyl-4-chloro-1-{[2′-(1*H*-tetrazol-5-yl)-4-biphenylyl]methyl}-1*H*-imidazol-5-yl)methanol, LOS). The structural formula of losartan was drawn with the use of the ACD/ChemSketch ver. 2018.2.1 program.

The increasing occurrence of obesity related to, i.a., the overuse of saturated fatty acids in the diet is a predisposing factor to the appearance of metabolic syndrome, which significantly increases the risk of type 2 diabetes and cardiovascular disease in adults [16]. Its main components, apart from obesity, are primarily arterial hypertension, insulin resistance, and atherogenic dyslipidemia. Losartan (LOS, Figure 2) is one of the significant drugs used in the regulation of arterial hypertension as well as in the treatment for chronic heart failure and the prevention of cardiovascular diseases in order to reduce the risk of a stroke in patients with hypertension and left ventricular hypertrophy. As a selective and competitive, nonpeptide angiotensin II (AII) receptor antagonist, LOS blocks the vasoconstrictor and aldosterone-secreting effects of angiotensin II [17].

The aim of this study was to evaluate the effect of fatty acid (FA) mixtures with different saturated (PA—palmitic acid, MYR—myristic acid, and SA—stearic acid) and unsaturated (OA—oleic acid and LA—linoleic acid) fatty acids on the affinity of losartan for glycated human serum albumin—simulating the state of diabetes in the body. Individual fatty acid mixtures corresponded to the FA content in physiological state and in various clinical states proceeding with increased concentrations of saturated (FA_S_) and unsaturated (FA_US_) acids. The binding properties of glycated albumin in the presence fatty acids and conformational changes of glycated human serum albumin were studied based on the quantitative analysis using absorption (UV-Vis) and fluorescence spectroscopy. 

As has been well described in the literature, both UV-Vis and fluorescence spectroscopy, mainly the quenching of biomacromolecules fluorescence method, are very helpful in protein–ligand and protein–ligand–ligand interactions due to their high sensitivity, rapidity, and ease of implementation [18,19,20,21,22]. Fluorescence measurements can provide some information about the binding of small molecules to proteins, such as the binding mechanism, binding mode, binding constants, and binding sites, and are useful in drug development in the early stage of research [19].

The research regarding the influence of fatty acids on the structure and binding properties of glycated human albumin, which simulates the states of diabetes in the body, is important from the scientific point of view because the conformational transformation of the most important transport protein—serum albumin—due to the many modifications that affect its stability and biological activity. This protein should be constantly monitored, especially in diseases and in the elderly. Monitoring the concentration of the drug–free fraction can help with optimizing pharmacotherapy as well as increase its effectiveness and avoid side effects.

## 2. Results and Discussion

### 2.1. The Interaction of Losartan with Glycated Human Serum Albumin in the Absence and in the Presence of Fatty Acids

Based on the emission fluorescence spectra of glycated, defatted (af)gHSA and glycated in the presence of fatty acids (af)gHSA_phys_, (af)gHSA_4S_, (af)gHSA_8S_, (af)gHSA_4US_, and (af)gHSA_8US_ albumin (5 × 10^−6^ mol∙L^−1^) (data not shown), an increase in the losartan (LOS) concentration (5 × 10^−6^ mol∙L^−1^–5 × 10^−5^ mol∙L^−1^) in ligand–albumin systems causes a gradual decrease in the macromolecule fluorescence intensity. According to Stryer theory, the observed effect may be associated with the quenching fluorescence of excited fluorophores (tryptophanyl residue (Trp-214) and tyrosyl residues (Tyrs)) of glycated human albumins by the losartan molecule, which was found in no more than 10 nm proximity [18]. 

This distance makes it possible to transfer energy to the ligand molecule. In addition, in the LOS-(af)gHSA system (from a 0:1 to 10:1 molar ratio), after excitation at λ_ex_ = 275 nm and λ_ex_ = 295 nm, the shift in the defatted albumin fluorescence emission band towards shorter waves (blue shift) by 13 nm (Δλ_max_ = 326–313 nm) and 2 nm (Δλ_max_ = 337–335 nm) relative to the spectrum of the ligand-free albumin has been observed. The hypsochromic shift of maximum albumin fluorescence indicates the formation of a hydrophobic environment around the tryptophanyl (Trp-214) and residues tyrosyl (Tyrs) of (af)gHSA due to the interaction of LOS with albumin. 

The shift in the fluorescence emission band of albumin relative to the albumin spectrum in the presence of LOS (Δλ_max_) smaller for LOS-(af)gHSA_phys_ (Δλ_max_ = 324–313 nm), LOS-(af)gHSA_4S_ (Δλ_max_ = 324–316 nm), LOS-(af)gHSA_8S_ (Δλ_max_ = 323–318 nm), LOS-(af)gHSA_4US_ (Δλ_max_ = 323–318 nm), and LOS-(af)gHSA_8US_ (Δλ_max_ = 322–320 nm) systems than for the LOS-(af)gHSA system at the excitation wavelength λ_ex_ = 275 nm, may indicate less variability of the hydrophobic/hydrophilic properties of LOS binding site as a result of the content of fatty acids in the structure of albumin (near the Trp-214 residue in subdomain IIA and Tyrs residues in subdomains IB, IIB, IIA, and IIIA). 

At the excitation λ_ex_ = 295 nm, no shift has been recorded. Lakowicz explained that the emission of indole Trp-214 may be blue shifted if the group is buried within a native protein, and its emission may shift to longer wavelengths (red shift) when protein is unfolded [19]. Similarly, as in our previous work, the presence of acetohexamide (AH)—a drug with hypoglycemic activity and a sulfonylurea derivative of the first generation—also caused a blue shift of glycated human serum albumin in the absence of FA (af)gHSA spectra in AH-(af)gHSA [23].

The blue shift of maximum albumin fluorescence (Δλ_max_) caused by the presence of losartan indicates the possibility of hydrophobic interactions between the aromatic rings of the LOS molecule and aromatic amino acid rings of the hydrophobic albumins cavity within IIA (Trp-214, Tyr-263) or/and IB (Tyr-138, Tyr-140, Tyr-148, Tyr-150, andTyr-161), IIB (Tyr-319, Tyr-332, Tyr-334, Tyr-341, Tyr-353, and Tyr-370) and IIIA (Tyr-401, Tyr-411, Tyr-452, and Tyr-497) subdomains [19,24]. 

Moeinpour et al., using a molecular dynamics simulation technique, also studied the interaction between losartan and glycated human serum albumin (gHSA). Based on the results visualized by Ligplus and Autodock (Figure 11 from [25]), they concluded that LOS was located within the hydrophobic binding pocket of gHSA, and several phenyl groups of the drug interacted with the Glu-348, Glu-345, Val-346, Lys-373, Leu-369, Phe-349, Lys-364, Asp-372, Glu-360, and Asn-361 residues of subdomain IIB of gHSA through hydrophobic interaction. 

Contrary to the study of LOS interaction with HSA, the specific hydrogen bonding interaction observed between the NH group of LOS and Asn-391 residue of albumin has not been identified. The environment of subdomain IIB, fatty acid high- (FA4), and low- (FA3, FA6, and FA7) affinity binding site, is likely the place where losartan can be located, and these sites could affect the binding. 

Moreover, by the use of multiple spectroscopic methods, Moeinpour et al. also observed a blue shift of HSA maximum wavelength (fatty-acid-free human serum albumin), as well as its glycated form (gHSA) with an increasing amount of losartan [25]. This effect explained that the chromophore of HSA and gHSA was found to be directed towards more hydrophobic environments, and the conformation of the proteins was changed by the presence of the drug.

**Figure 3:**
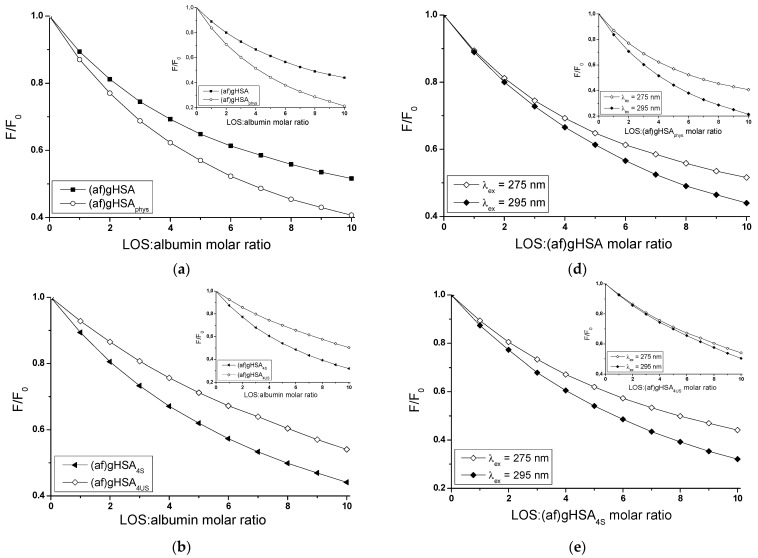

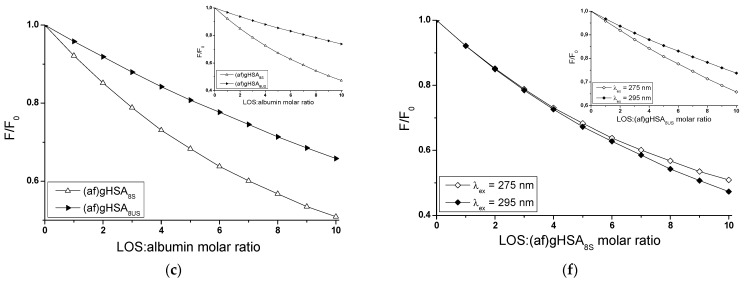
Fluorescence quenching of (**a**) (af)gHSA, (af)gHSA_phys_, (**b**) (af)gHSA_4S_, (af)gHSA_4US_, (**c**) (af)gHSA_8S_, (af)gHSA_8US_ complexed with LOS (5 × 10^−6^ mol∙L^−1^–5 × 10^−5^ mol∙L^−1^), λ_ex_ = 275 nm (in the main view), λ_ex_ = 295 nm (in the insert) and fluorescence quenching of (**d**) LOS-(af)gHSA, (**e**) LOS-(af)gHSA_4S_, (**f**) LOS-(af)gHSA_8S_ system (in the main view) and (**d**) LOS-(af)gHSA_phys_, (**e**) LOS-(af)gHSA_4US_, and (**f**) LOS-(af)gHSA_8US_ system (in the insert) for λ_ex_ = 275 nm and λ_ex_ = 295 nm; the albumin concentration was 5 × 10^−6^ mol∙L^−1^; the error bars are smaller than the symbols.

Fluorescence quenching curves present 5 × 10^−6^ mol∙L^−1^ glycated human serum albumin (af)gHSA in the absence and in the presence of fatty acids ((af)gHSA_phys_, (af)gHSA_4S_, (af)gHSA_8S_, (af)gHSA_4US_, and (af)gHSA_8US_) fluorescence quotient in the absence (F_0_) and in the presence of LOS (5 × 10^−6^ mol∙L^−1^–5 × 10^−5^ mol∙L^−1^) (F) in the function of the drug:albumin molar ratio, λ_ex_ = 275 nm and λ_ex_ = 295 nm (Figure 3).

The course of albumin fluorescence quenching curves illustrates the reduction in fluorescence intensity of human serum albumin (af)gHSA in the absence of fatty acids and with fatty acids ((af)gHSA_phys_, (af)gHSA_4S_, (af)gHSA_8S_, (af)gHSA_4US_, and (af)gHSA_8US_)) with the increase of losartan concentration in LOS-glycated albumin system (Figure 3a–c, in the main view and in the insert). The presence of fatty acids affects the ability of losartan to quench albumin fluorescence. 

Table 1 shows the percentage of fluorescence quenching (af)gHSA and (af)gHSA_phys_, (af)gHSA_4S_ and (af)gHSA_4US_, (af)gHSA_8S_ and (af)gHSA_8US_ (5 × 10^−6^ mol∙L^−1^) for the highest concentration of LOS (5 × 10^−5^ mol∙L^−1^). The data collected in Table 1 show that the strongest quenching of albumin fluorescence in the presence of losartan with the increase of concentration was in the range of 59.34% and 77.80% for (af)gHSA_phys_ at λ_ex_ = 275 nm and λ_ex_ = 295 nm, respectively. 

This means that losartan is sufficiently close to protein tryptophanyl or/and tyrosyl residues (not more than 10 nm) and has the strongest affinity for (af)gHSA_phys_ molecule than for (af)gHSA and (af)gHSA_4S_, (af)gHSA_8S_, (af)gHSA_4US_, and (af)gHSA_8US_. A stronger fluorescence quenching for (af)gHSA_phys_ than (af)gHSA (Figure 3a), for (af)gHSA_4S_ than (af)gHSA_4US_ (Figure 3b), for (af)gHSA_8S_ than (af)gHSA_8US_ (Figure 3c), at both excitation wavelengths λ_ex_ = 275 nm (in the main view) and λ_ex_ = 295 nm (in the insert), has been observed. 

This demonstrates a higher losartan ability to absorb energy from excited fluorophores of albumin in the presence of fatty acids at physiological concentration ((af)gHSA_phys_) than from defatted albumin ((af)gHSA) and from albumin containing four times ((af)gHSA_4S_) and eight-times ((af)gHSA_8S_) higher amounts of saturated than unsaturated ((af)gHSA_4US_, (af)gHSA_8US_) fatty acids. This phenomenon is a result of conformational changes caused by the presence of fatty acids at physiological concentration or lower contents of saturated and unsaturated fatty acids.

As previously mentioned, after the excitation of albumin at λ_ex_ = 295 nm, the observed emission of fluorescence comes almost exclusively from a tryptophanyl residue (Trp-214), while, for λ_ex_ = 275 nm, this is from both Trp-214 and tyrosyl residues (Tyrs). The comparison between fluorescence quenching curves of glycated, defatted ((af)gHSA), and glycated in the presence of fatty acids ((af)gHSA_phys_, (af)gHSA_4S_, (af)gHSA_8S_, (af)gHSA_4US_, and (af)gHSA_8US_) albumins in the presence of losartan at λ_ex_ = 275 nm and λ_ex_ = 295 nm indicated the fluorophores involved in the interaction with the drug. 

An almost identical course of albumin fluorescence quenching curves in LOS-(af)gHSA_4US_ (Figure 3e, in the insert) and LOS-(af)gHSA_8S_ (Figure 3f, in the main view) system at λ_ex_ = 275 nm and λ_ex_ = 295 nm (almost a 4% difference in quenching of the intrinsic albumin fluorescence, Table 1) indicates the contribution of Trp-214 or its environment and a negligible contribution of Tyrs in the interaction of LOS with both, (af)gHSA_4US_ and (af)gHSA_8S_ in the environment of binding site. The human serum albumin contains only one tryptophanyl group. 

It can be argued that LOS interacts with albumin containing four-times higher amounts of unsaturated ((af)gHSA_4US_) and eight-times higher amounts of saturated ((af)gHSA_8S_) fatty acids in relation to the physiological concentration mainly in subdomain IIA, but the possibility of LOS interaction with albumin other sites cannot be excluded. 

Differences in the course of quenching fluorescence at both excitation wavelengths λ_ex_ = 275 nm and λ_ex_ = 295 nm (almost 8% in LOS-(af)gHSA (Figure 3d, in the main view) and LOS-(af)gHSA_8US_ (Figure 3f, in the insert), 12% in LOS-(af)gHSA_4S_ (Figure 3e, in the main view), and more than 18% in LOS-(af)gHSA_phys_ (Figure 3d, in the insert) system (Table 1)), indicate the simultaneous participation of the Trp-214 residue located in subdomain IIA and Tyrs residues located in the IIA, IB, and IIB and subdomains in the interaction of LOS with albumin at the appropriate binding site. As reported in the literature, tyrosyl residues in position 401 (Tyr-401) and 411 (Tyr-411) located in the IIIA subdomain of albumin play a major role in drug binding [19,26]. The fluorescence quenching technique is not sufficient to indicate which Tyrs moieties are involved in LOS binding.

**Figure 4:**
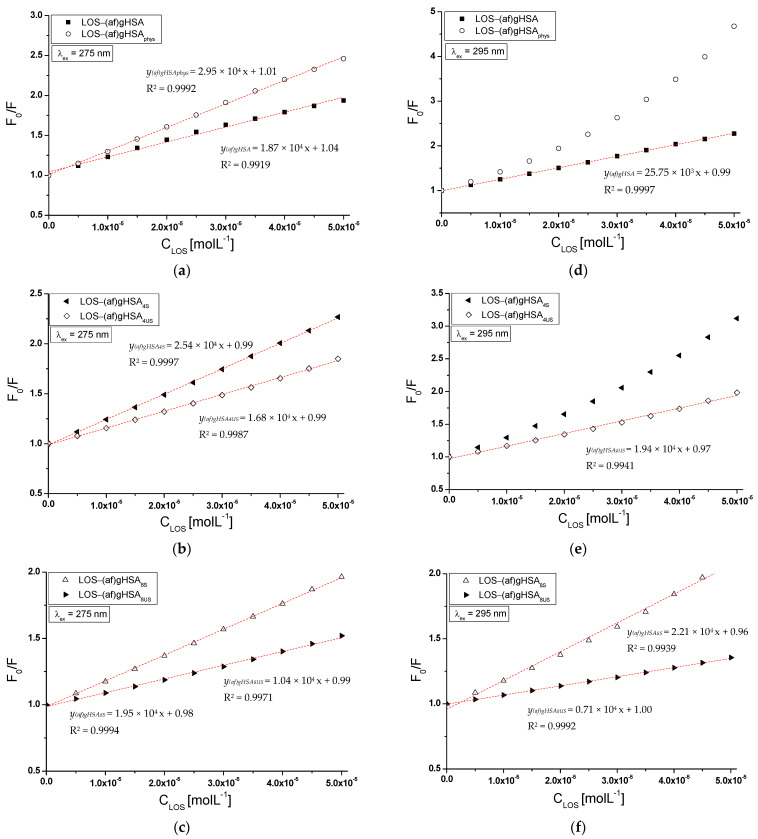
The Stern–Volmer curves for (**a**,**d**) LOS-(af)gHSA and LOS-(af)gHSA_phys_, (**b**,**e**) LOS-(af)gHSA_4S_ and LOS-(af)gHSA_4US_, (**c**,**f**) LOS-(af)gHSA_8S_ and LOS-(af)gHSA_8US_; (**a**–**c**) λ_ex_ = 275 nm, (**d**–**f**) λ_ex_ = 295 nm; the error bars are smaller than the symbols.

The mechanism of losartan interaction with albumin can be determined on the basis of Stern–Volmer curves (Equation (2)). Based on the data obtained from glycated, defatted (af)gHSA and glycated in the presence of fatty acids (af)gHSA_phys_, (af)gHSA_4S_, (af)gHSA_8S_, (af)gHSA_4US_, and (af)gHSA_8US_ albumin in the presence of LOS, the Stern–Volmer curves were plotted, λ_ex_ = 275 nm (Figure 4a–c) and λ_ex_ = 295 nm (Figure 4d–f). The dashed lines indicate a model rectilinear course of Stern–Volmer dependence (F_0_/F = *f*([C_LOS_]).

The Stern–Volmer curves obtained for the LOS-(af)gHSA, LOS-(af)gHSA_4S_, and LOS-(af)gHSA_8S_ system show a different course of curves plotted for the LOS-(af)gHSA_phys_ (Figure 4a,d), LOS-(af)gHSA_4US_ (Figure 4b,e), and LOS-(af)gHSA_8US_ (Figure 4c,f) systems at both excitations λ_ex_ = 275 nm and λ_ex_ = 295 nm. Higher fluorescence quenching (F_0_/F) for the whole range of losartan concentrations occurred for glycated albumin (af)gHSA_phys_ in the presence of physiologically fatty acids and albumin containing four- and eight-times higher amounts of saturated fatty acids ((af)gHSA_4S_ and (af)gHSA_8S_) in relation to the physiological concentration compared to the F_0_/F values obtained for glycated, defatted (af)gHSA albumin and containing four- and eight-times higher amounts of unsaturated fatty acids ((af)gHSA_4US_, (af)gHSA_8US_) in relation to the physiological concentration (Figure 4). 

Positive deviation (deviation in the OY direction) from the rectilinear relationship F_0_/F = *f*[C_LOS_] for LOS-(af)gHSA_phys_ (above the LOS concentration 2 × 10^−5^ mol∙L^−1^) (Figure 4d) and LOS-(af)gHSA_4S_ (above the LOS concentration 2.5 × 10^−5^ mol∙L^−1^) (Figure 4e) system at λ_ex_ = 295 nm, indicates the occurrence of both dynamic and static quenching fluorescence of (af)gHSA_phys_ and (af)gHSA_4S_ albumin by losartan. During dynamic quenching, the ligand penetrates the environment of the macromolecule, and fluorescence quenching is caused by the collision of the quencher molecule and the fluorophore (Trp-214) of albumin in excitation state. 

On the other hand, static quenching leads to a reduction in fluorescence intensity when the ligand binds to the fluorophore molecule in its basic state (unexcited), reducing the population of excitable fluorophores [20]. The existence of dynamic and static quenching of human serum albumin fluorescence was obtained in our previous studies when the influence of piracetam (as a potential glycation inhibitor) on gliclazide-glycated albumin interaction was analyzed [27]. 

The linear F_0_/F = *f*[C_LOS_] relationship for the other systems (Figure 4), indicates a dynamic or static mechanism of macromolecule fluorescence quenching in the environment of subdomains containing amino acid residues that are involved in the formation of the ligand–albumin complex. Moreover, the order of fluorescence quenching rate constants k_q_ equals to 10^12^ determined for LOS-glycated albumin system clearly indicates a static fluorescence quenching mechanism (Table 2), while according to Lakowicz, when the maximum value of the k_q_ constant in the aqueous solution equals to 1 × 10^10^ (mol^−1^∙L∙s^−1^), the dynamic fluorescence quenching mechanism occurs [19].

**Figure 5:**
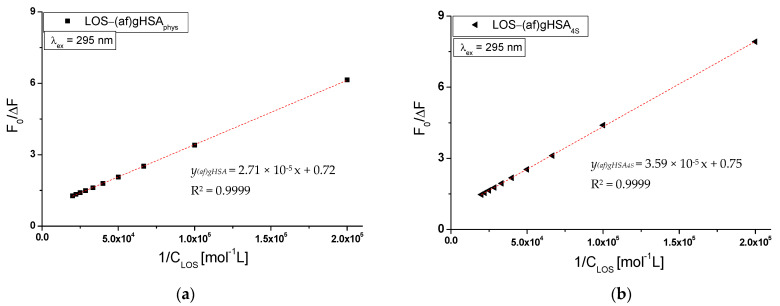
The Stern–Volmer curves modified by Lehrer for (**a**) LOS-(af)gHSA_phys_ and (**b**) LOS-(af)gHSA_4S_ system; λ_ex_ = 295 nm; the error bars are smaller than the symbols.

From the F_0_/F = *f*[C_LOS_] relationship for a system with a linear course of the Stern–Volmer curve, the Stern–Volmer constants K_SV_, the bimolecular quenching rate constants k_q_ and maximum available fluorescence fraction f_a_ of all albumin fluorophores were determined. Quenching parameters (K_SV_, k_q_ = K_SV_/τ_0_) and f_a_ for a system with non-linear Stern–Volmer relationship (LOS-(af)gHSA_phys_ (Figure 4d) and LOS-(af)gHSA_4S_ (Figure 4e) for λ_ex_ = 295 nm) were determined from F_0_/∆F = *f*(1/[C_LOS_]) relationship represented by Stern–Volmer equation modified by Lehrer (Equation (3)) [21]. The plot of F_0_/∆F vs. 1/[C_LOS_] is found to be linear with the intercept on the ordinate (Figure 5a,b). The reciprocal of the intercept gives the value of f_a_ while the intercept/slope gives the value of the Stern–Volmer constants K_SV_. The obtained results have been collected in Table 2.

The Stern–Volmer constant is used to assess the availability of the quencher to the excited fluorophore. The growth of K_SV_ value is associated with the increase of ligand molecule availability to the macromolecule and the formation of the complex in an excited state. As can be seen in the Table 2, the higher values of K_SV_ constant obtained for the LOS-(af)gHSA_phys_ system compared to K_SV_ obtained for LOS-(af)gHSA indicate the location of losartan molecules closer to the fluorophores of glycated, fatted by fatty acids physiological mixture albumin ((af)gHSA_phys_) than glycated, defatted albumin (af)gHSA fluorophores. 

The presence of fatty acids physiological mixture in glycated human serum albumin probably makes formation of LOS-(af)gHSA_phys_ complex easier than the absence of fatty acids in the system (especially when the observed emission of fluorescence comes from both Trp-214 and tyrosyl residues (Tyrs)). The Stern–Volmer values and biomolecular quenching rate constants obtained for LOS-(af)gHSA_4S_ and LOS-(af)gHSA_8S_ are higher than K_SV_ and k_q_ values obtained for LOS-(af)gHSA_4US_ and LOS-(af)gHSA_8US_ (λ_ex_ = 275 nm and λ_ex_ = 295 nm). 

Moreover, a two-fold increase in the amount of saturated fatty acids in the LOS–albumin system resulted in 23% decrease of K_SV_ constant for λ_ex_ = 275 nm and only 6% increase Ksv for λ_ex_ = 295 nm. On the other hand, a two-fold increase in the amount of unsaturated fatty acids in the LOS–albumin system caused 38% and 63% decreases in K_SV_ for λ_ex_ = 275 nm and λ_ex_ = 295 nm, respectively. These results indicate that LOS molecules locate at different distances to fluorophores of glycated albumin containing various amounts of saturated and unsaturated fatty acids. In addition, it can be seen that the availability of albumin (af)gHSA_phys_ and (af)gHSA_4S_ fluorophores (especially Trp-214 residues) for individual LOS binding sites is significantly facilitated (Table 2).

**Figure 6:**
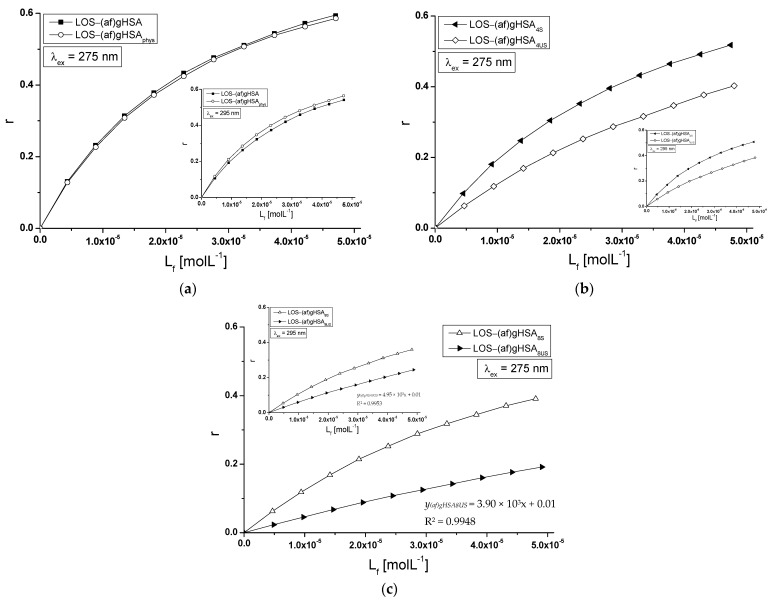
Binding isotherms of (**a**) (af)gHSA, (af)gHSA_phys_, (**b**) (af)gHSA_4S_, (af)gHSA_4US_, and (**c**) (af)gHSA_8S_, (af)gHSA_8US_ at 5 × 10^−6^ mol∙L^−1^ concentration with LOS at 5 × 10^−6^–5 × 10^−5^ mol∙L^−1^ concentration, λ_ex_ = 275 nm (in the main view), λ_ex_ = 295 nm (in the insert); the error bars are smaller than the symbols.

To determine the nature of the interaction of losartan with glycated, defatted (af)gHSA and glycated in the presence of fatty acids albumin (af)gHSA_phys_, (af)gHSA_4S_, (af)gHSA_8S_, (af)gHSA_4US_, and (af)gHSA_8US_, binding isotherms were plotted in the LOS-(af)gHSA and LOS-(af)gHSA_phys_ (Figure 6a), LOS-(af)gHSA_4S_ and LOS-(af)gHSA_4US_ (Figure 6b), and LOS-(af)gHSA_8S_ and LOS-(af)gHSA_8US_ (Figure 6c) systems, λ_ex_ = 275 nm (Figure 6, in the main view) and λ_ex_ = 295 nm (Figure 6, in the insert).

Similarly, as in our previous paper [28], where the interaction of tolbutamide and losartan with human serum albumin in hyperglycemia states were studied, a non-linear relationship r = *f*([L_f_]) was observed (Figure 6). The non-linear shape of the binding isotherms obtained for LOS-(af)gHSA and LOS-(af)gHSA_phys_ (Figure 6a), LOS-(af)gHSA_4S_ and LOS-(af)gHSA_4US_ (Figure 6b), and LOS-(af)gHSA_8S_ (Figure 6c) complexes indicates the mixed (specific and non-specific) nature of losartan interaction with macromolecules. 

This means that losartan binds not only to its specific binding sites in glycated, defatted and in the presence of fatty acids albumin but also non-specifically interacts with the hydrophobic fragments of its surface [29]. However, the shape of the binding isotherms for glycated human serum albumin with fatty acids containing eight-times more unsaturated fatty acids in relation to the physiological value, indicates the occurrence of only the non-specific nature of losartan binding to (af)gHSA_8US_ (Figure 6c). 

Specific binding is characterized by high affinity and low binding capacity, while non-specific binding is characterized by low affinity and unlimited drug binding capacity [29]. Regardless of the course of binding isotherms (Figure 6), the losartan–glycated albumin interaction is likely characterized by a specific type of binding because, in physiological environments, the drug:albumin molar ratio is much smaller than 1:1 and equals to 1:500.

**Figure 7:**
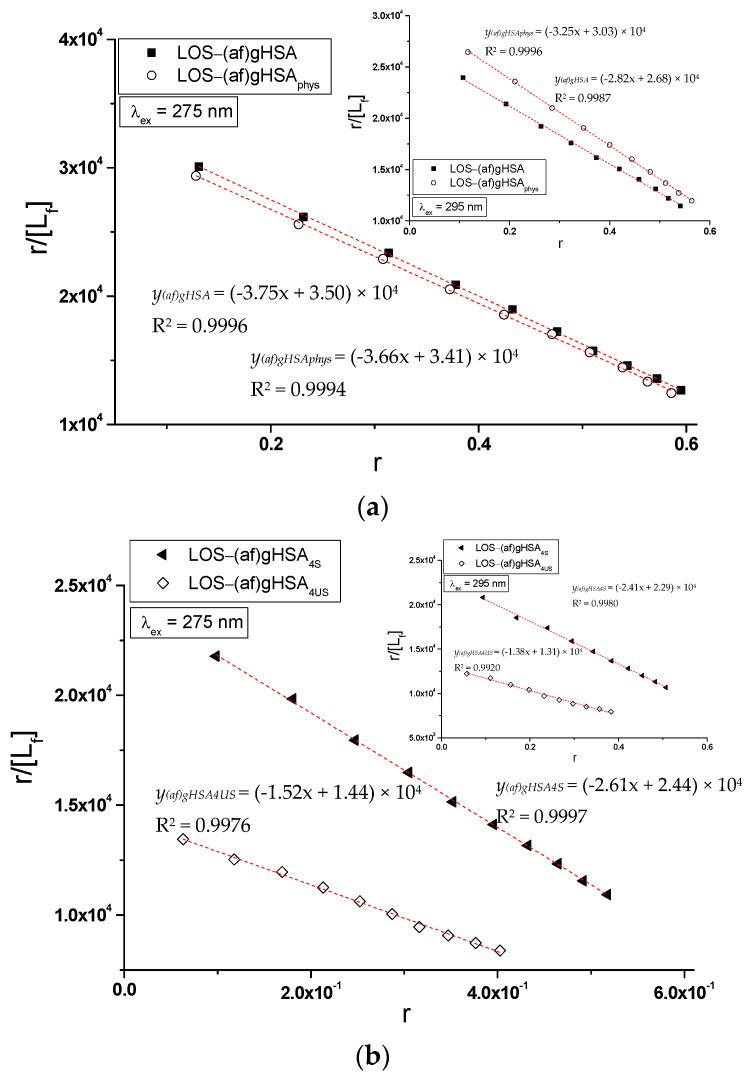

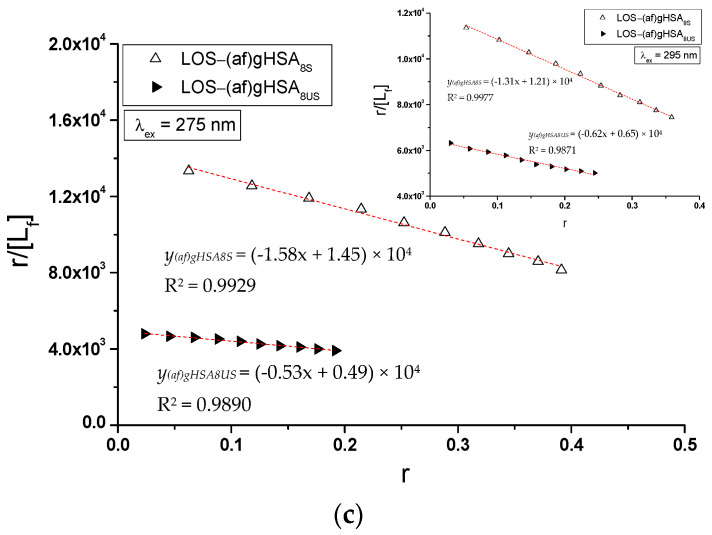
The Scatchard curves of r/[L_f_] vs. r for (**a**) LOS-(af)gHSA and LOS-(af)gHSA_phys_, (**b**) LOS-(af)gHSA_4S_ and LOS-(af)gHSA_4US_, and (**c**) LOS-(af)gHSA_8S_ and LOS-(af)gHSA_8US_ systems, λ_ex_ = 275 nm (in the main view), λ_ex_ = 295 nm (in the insert); the error bars are smaller than the symbols.

**Figure 8:**
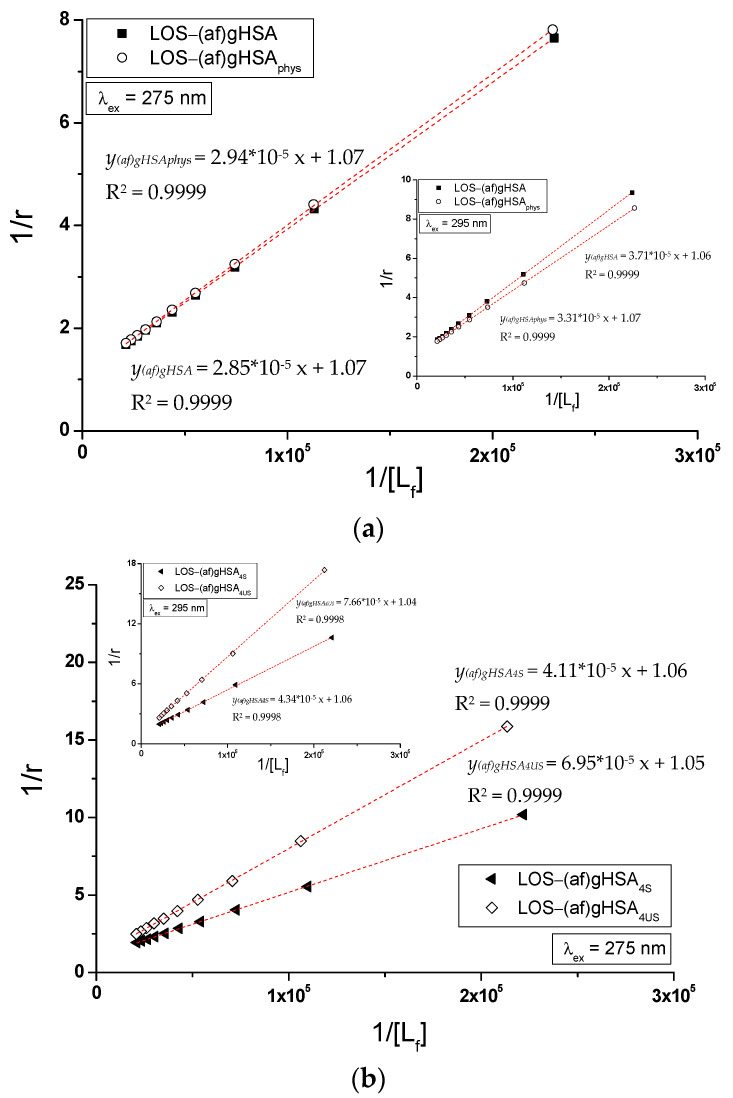

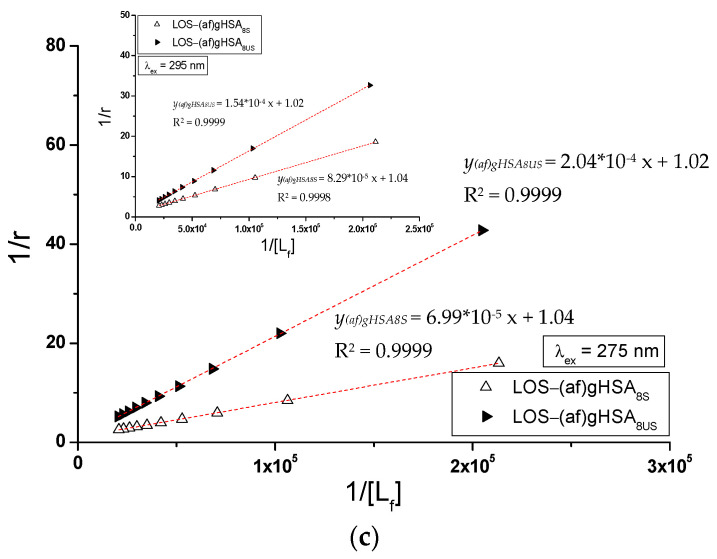
The Klotz curves of 1/r vs. 1/[L_f_] for (**a**) LOS-(af)gHSA and LOS-(af)gHSA_phys_, (**b**) LOS-(af)gHSA_4S_ and LOS-(af)gHSA_4US_, and (**c**) LOS-(af)gHSA_8S_ and LOS-(af)gHSA_8US_ systems, λ_ex_ = 275 nm (in the main view), λ_ex_ = 295 nm (in the insert); the error bars are smaller than the symbols.

There are many methods for the calculation of association constant (K_a_) that characterizes the stability of formed drug–albumin complex for the determination the number of drug molecules (*n*) associated with one albumin molecule at equilibrium, or for the prediction an existence of one or more independent classes of binding sites. In the present work, specific binding of losartan to glycated human serum albumin in LOS-(af)gHSA, LOS-(af)gHSA_phys_, LOS-(af)gHSA_4S_, LOS-(af)gHSA_4US_, LOS-(af)gHSA_8S_, and LOS-(af)gHSA_8US_ complexes has been quantitatively characterized using the association constant K_a_ calculated based on the Scatchard (the dependence of r/[L_f_] on r, Equation (4), Figure 7) and the Klotz (the dependence of 1/r on 1/[L_f_], Equation (5), Figure 8) equations. 

**Figure 9:**
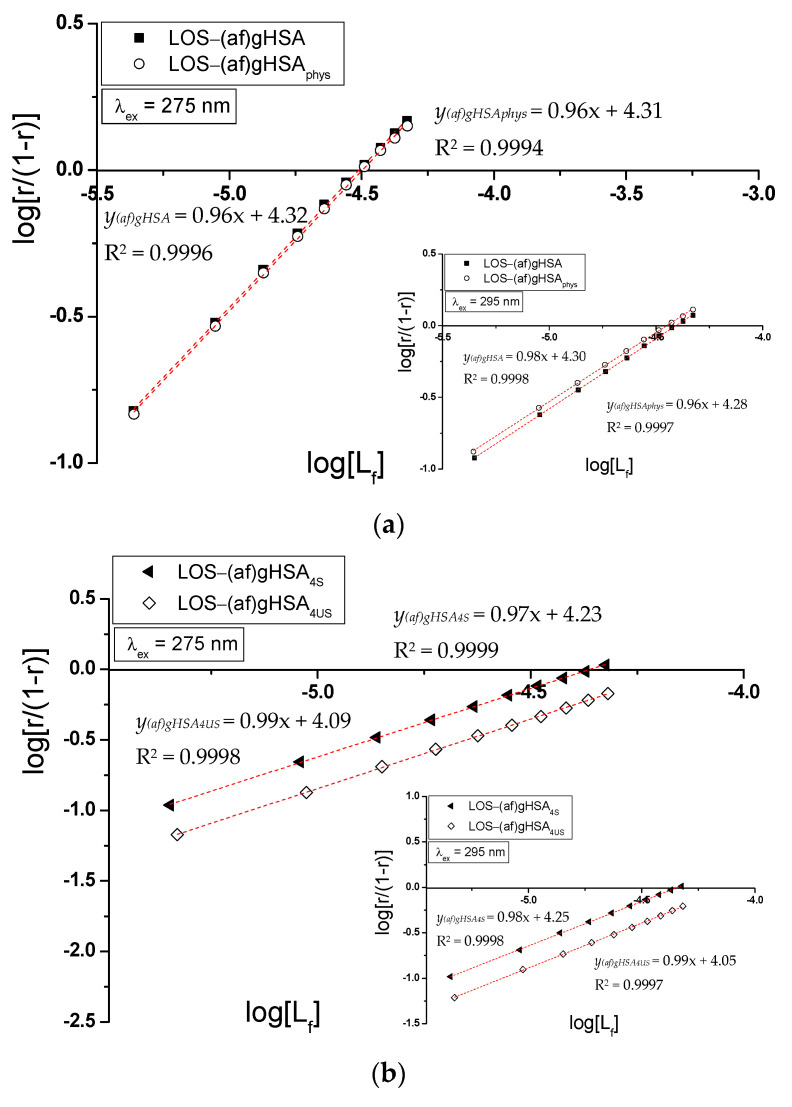

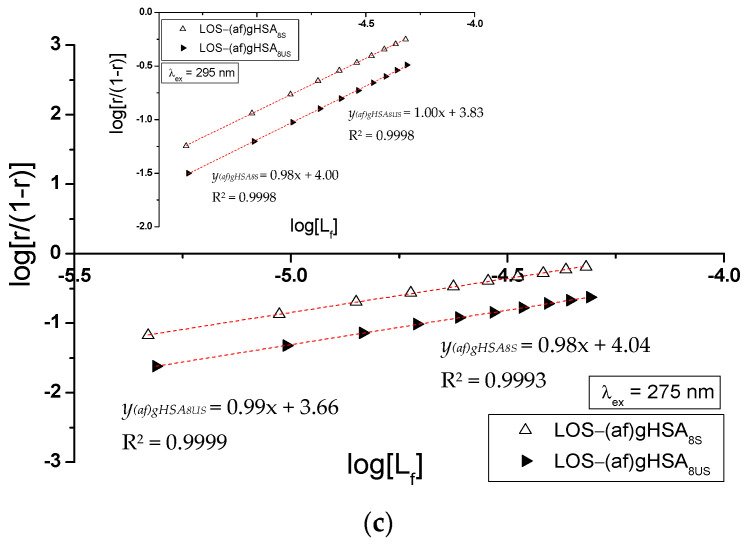
The Hill curves of log[r/(1-r)] vs. log[L_f_] for (**a**) LOS-(af)gHSA and LOS-(af)gHSA_phys_, (**b**) LOS-(af)gHSA_4S_ and LOS-(af)gHSA_4US_, and (**c**) LOS-(af)gHSA_8S_ and LOS-(af)gHSA_8US_ systems, λ_ex_ = 275 nm (in the main view), λ_ex_ = 295 nm (in the insert); the error bars are smaller than the symbols.

In the Scatchard equation, the concentration of the bound ligand to the protein is the independent variable, while, in the Klotz equation, the independent variable is the reciprocal of the free ligand fraction. To study the possible cooperation of losartan binding to the macromolecule, the Hill interaction factors (n_H_) were determined by the use of the Hill equation (the dependence of log[r/(1 − r)] on log[L_f_], Equation (6), Figure 9). The number of losartan molecules (*n*) forming a complex with one molecule of (af)gHSA, (af)gHSA_phys_, (af)gHSA_4S_, (af)gHSA_4US_, (af)gHSA_8S_, and (af)gHSA_8US_ at equilibrium state for a specific class of binding sites was also obtained. The binding parameters (K_a_, *n*) and Hill n_H_ coefficient (interaction factor) are summarized in Table 3.

The straight-line Scatchard (Figure 7), Klotz (Figure 8) and Hill (Figure 9) plots indicate the presence of one independent class of losartan binding sites in the albumin (af)gHSA, (af)gHSA_phys_, (af)gHSA_4S_, (af)gHSA_8S_, (af)gHSA_4US_, and (af)gHSA_8US_ structure (or one binding site). The course of binding isotherms, which determine the binding specificity of ligand to albumin, for LOS-(af)gHSA_8US_ system is linear (Figure 6c). The straight-line relationship of r = *f*([L_f_]) Taira and Terada [29] explained by the non-specific interaction of ligand with the hydrophobic fragments of macromolecule surfaces. 

The association constants K_a_ determined from the Scatchard and the Klotz relationships for the complexes LOS-(af)gHSA_8US_ for λ_ex_ = 275 nm and λ_ex_ = 295 nm prove the specific nature of losartan binding within the albumin (Table 3). For LOS-(af)gHSA complex, the association constants K_a_ are the same (for λ_ex_ = 275 nm) and not much lower (for λ_ex_ = 295 nm) than the constants K_a_ values obtained for losartan-(af)gHSA_phys_ complex (Table 3), which indicates that LOS has the same affinity for (af)gHSA and (af)gHSA_phys_ albumin binding sites. 

For the LOS–albumin complex with a two-times greater amount of saturated ((af)gHSA_8S_) and unsaturated ((af)gHSA_8US_) fatty acids, the K_a_ constants are smaller than those obtained for the LOS-(af)gHSA_4S_ and LOS-(af)gHSA_4US_ for λ_ex_ = 275 nm and 295 nm (Table 3). This means that a higher concentration of both saturated and unsaturated fatty acids in glycated albumin reduces the stability of the complex formed with losartan. For LOS-(af)gHSA, LOS-(af)gHSA_phys_, LOS-(af)gHSA_4S_, LOS-(af)gHSA_8S_, LOS-(af)gHSA_4US_, and LOS-(af)gHSA_8US_ complexes, an average of one ligand molecule binds to one albumin molecule (*n* ≈ 1). 

The Hill interaction coefficient n_H_ equals unity (n_H_ ≈ 1) and indicates a lack of cooperativity in the binding of LOS to albumins in the vicinity of Trp-214 and Tyrs residues. This is the same value of n_H_ that we obtained in our previous work [23] when we determined the Hill interaction coefficient for acetohexamide–albumin complex with four- and eight-fold higher unsaturated and eight-fold higher saturated fatty acids amount compared to physiological value.

Based on the in vitro results, the fatty acids affect losartan binding to glycated human serum albumin. It can be assumed that, under conditions of abnormal fat content in the body, the pharmacokinetics of the drug may be disturbed. It is noteworthy that during a treatment with losartan it is important to control the amount of fatty acids supplied to the body with diet and/or in the form of supplements. Stronger binding of LOS to albumin weakens its therapeutic effect; however, on the other hand, the free drug fraction has potentially toxic side effects that can be dangerous to the patient’s health. The research suggests the need for individual dose selection, especially for the obese patients with chronic diseases.

### 2.2. Structural Modification of Glycated Human Serum Albumin Caused by Fatty Acids

The physicochemical and biological properties of proteins are directly dependent on their spatial structure. It is for this reason that studies that allow us to observe conformational changes in protein caused by various modifications are important. A number of structural modifications, especially in the tertiary confirmation of human serum albumin, are attributed to, e.g., protein glycation [30]. 

In this part of the study, we examined whether fatty acids cause additional conformational changes in the tertiary structure of glycated albumin, which simulates diabetes in the body. It is of key importance in planning therapy because the strength and nature of the drug’s interactions with its main distributor may change in the presence of coexisting diabetes and obesity (Section 2.1). 

Although circular dichroism (CD) plays an important role in the study of protein folding as it allows the characterization of secondary and tertiary structure of proteins in native, unfolded and partially folded states [31], the CD spectra of the studied proteins were impossible to register due to the presence of the introduced fatty acids. Hence, to indicate changes in the tertiary structure of glycated albumin induced by the presence of fatty acids, fluorescence spectroscopy was used. 

For this purpose, we compared the emission and synchronous fluorescence spectra of glycated human serum albumin ((af)gHSA) in the absence and in the presence of fatty acids (FA) corresponding to the physiological FA composition in human blood ((af)gHSA_phys_) and in various clinical states, proceeding with increased concentrations of saturated ((af)gHSA_4S_ and (af)gHSA_8S_) and unsaturated FA ((af)gHSA_4US_ and (af)gHSA_8US_) compared to physiological values. Additionally, based on the Red Edge Excitation Shift (REES) analysis, the spatial organization of the Trp-214 molecules was determined.

**Figure 10:**
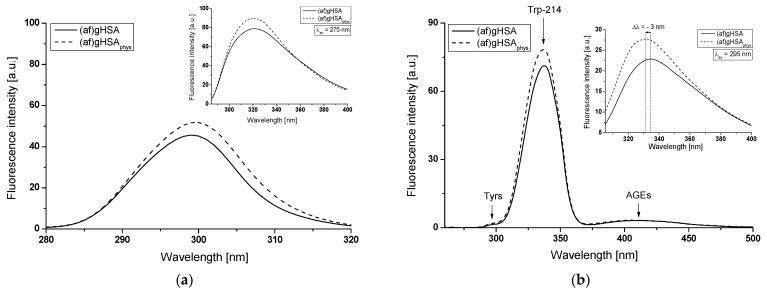
Main view: synchronous fluorescence spectra of (af)gHSA and (af)gHSA_phys_ at 5 × 10^−6^ mol∙L^−1^ concentration (**a**) Δλ = 15 nm (λ_ex_ = 265–305 nm) and (**b**) Δλ = 60 nm (λ_ex_ = 220–440 nm). Insert: comparison of (af)gHSA and (af)gHSA_phys_ emission fluorescence spectra excited at (**a**) λ_ex_ = 275 nm and (**b**) λ_ex_ = 295 nm; t = 37 °C.

**Figure 11:**
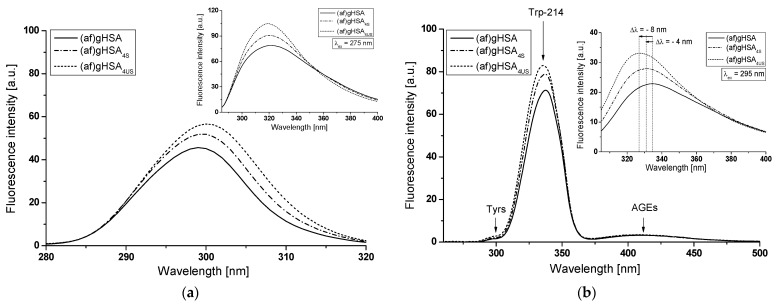
Main view: synchronous fluorescence spectra of (af)gHSA, (af)gHSA_4S_, (af)gHSA_4US_ at 5 × 10^−6^ mol∙L^−1^ concentration (**a**) Δλ = 15 nm (λ_ex_ = 265–305 nm) and (**b**) Δλ = 60 nm (λ_ex_ = 220–440 nm). Insert: comparison of (af)gHSA, (af)gHSA_4S_, (af)gHSA_4US_ emission fluorescence spectra excited at (**a**) λ_ex_ = 275 nm and (**b**) λ_ex_ = 295 nm; t = 37 °C.

**Figure 12:**
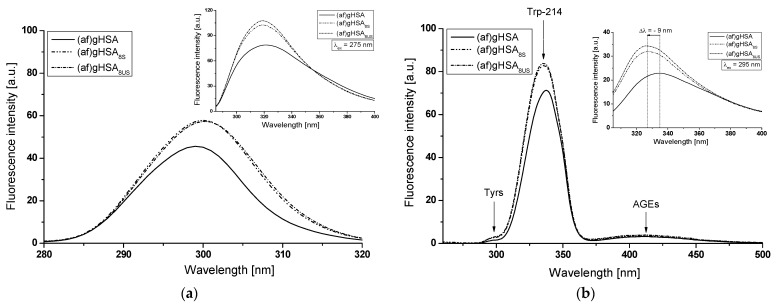
Main view: synchronous fluorescence spectra of (af)gHSA, (af)gHSA_8S_, (af)gHSA_8US_ at 5 × 10^−6^ mol∙L^−1^ concentration (**a**) Δλ = 15 nm (λ_ex_ = 265–305 nm) and (**b**) Δλ = 60 nm (λ_ex_ = 220–440 nm). Insert: comparison of (af)gHSA, (af)gHSA_8S_, (af)gHSA_8US_ emission fluorescence spectra excited at (**a**) λ_ex_ = 275 nm and (**b**) λ_ex_ = 295 nm; t = 37 °C.

Synchronous (Figure 10, Figure 11 and Figure 12, main view) and emission (Figure 10, Figure 11 and Figure 12, insert) fluorescence spectra of (af)gHSA and (af)gHSA_phys_ (Figure 10); (af)gHSA, (af)gHSA_4S_ and (af)gHSA_4US_ (Figure 11); (af)gHSA, (af)gHSA_8S_ and (af)gHSA_8US_ (Figure 12) were used to show the conformational changes in the environment of the tryptophanyl and tyrosine residues of glycated human serum albumin influenced by fatty acids. It is well known that the wavelength of 275 nm excites not only Trp-214 but also tyrosine residues and it is impossible to separately observe the fluorescence of these fluorophores. 

Synchronous fluorescence spectroscopy allows for separation of the emission spectra originating from the Trp-214 and Tyrs (as illustrated in Figure 10, Figure 11 and Figure 12b, main view), which results more specific information about the structure of the macromolecules. According to literature data [25,28,32], the synchronous fluorescence spectra were obtained considering the wavelength intervals Δλ = 60 nm and Δλ = 15 nm to evidence the Trp-214 and Tyrs, respectively (Δλ = λ_em_ − λ_ex_).

The fluorescence of human serum albumin fluorophores is sensitive to the changes of HSA tertiary structure and environmental properties. Slight structural changes in albumin near the Trp-214 and Tyrs residues affect the fluorescence intensity (F_max_) and position of maximum fluorescence (λ_max_) [33]. A blue shift of λ_max_ indicates that the Trp-214 and Tyrs residues are located in a more hydrophobic environment, while a red-shift of λ_max_ implies that the amino acid residues are in a polar environment and are more exposed to the solvent [34]. 

Using Δλ = 15 nm (Figure 10a, Figure 11 and Figure 12a, main view) and Δλ = 60 nm (Figure 10, Figure 11 and Figure 12b, main view) no changes were observed in the maximum emission wavelength of (af)gHSA and (af)gHSA_phys_ (Figure 10); (af)gHSA, (af)gHSA_4S_ and (af)gHSA_4US_ (Figure 11); (af)gHSA, (af)gHSA_8S_, and (af)gHSA_8US_ (Figure 12) Tyrs and Trp-214 residues (Table 4). This indicates the stability of both bands in the synchronous spectra of glycated human serum albumin in the absence (af)gHSA and in the presence of fatty acids (af)gHSA_phys_, (af)gHSA_4S_, (af)gHSA_8S_, (af)gHSA_4US_, and (af)gHSA_8US_. 

A lack of synchronous spectra shift (af)gHSA caused by the presence of fatty acids indicates no change in the polarity around Trp-214 and Tyr residues or/and a modification of the structure of glycated human serum albumin in the environment of other residues. On the other hand, the main characteristic of fatted human serum albumin emission fluorescence spectra excited at λ_ex_ = 295 nm (Figure 10, Figure 11 and Figure 12b, insert) is the blue shift maximum fluorescence for (af)gHSA_phys_ (Δλ = 3 nm, Figure 10b), for (af)gHSA_4S_ (Δλ = 4 nm, Figure 11b), for (af)gHSA_4US_ (Δλ = 8 nm, Figure 11b), and for (af)gHSA_8S_ and (af)gHSA_8US_ (Δλ = 9 nm, Figure 12b), Table 4. 

This phenomenon suggests that Trp-214 of glycated human serum albumin ((af)gHSA_phys_, (af)gHSA_4S_, (af)gHSA_8S_, (af)gHSA_4US_, and (af)gHSA_8US_) in the presence fatty acids are less exposed to the solvent than deffated macromolecule ((af)gHSA). The signal at λ_em_ ≅ 410 nm obtained in synchronous mode of (af)gHSA, (af)gHSA_phys_, (af)gHSA_4S_, (af)gHSA_4US_, (af)gHSA_8S_, and (af)gHSA_8US_ (Figure 10, Figure 11 and Figure 12, insert) indicates the presence of fluorescent AGEs–pentosidines and argyrimidines and or/ additional fluorophores created in albumin glycation [35]. 

The fluorescence intensity of both types of fluorophores in the (af)gHSA spectrum is lower than in the (af)gHSA_phys_, (af)gHSA_4S_, (af)gHSA_8S_, (af)gHSA_4US_, and (af)gHSA_8US_ spectra (Table 4). These results indicate an alteration of the glycated albumin tertiary structure by binding of fatty acids in the region of tryptophanyl and tyrosyl residues occurrence in subdomain IIA (Trp-214, Tyr-263), IB (Tyr-138, Tyr-140, Tyr-148, Tyr-150, and Tyr-161), IIB (Tyr-319, Tyr-332, Tyr-334, Tyr-341, Tyr-353, and Tyr-370), and IIIA (Tyr-401, Tyr-411, Tyr-452, and Tyr-497).

Red Edge Excitation Shift (REES) is an another method to directly monitor of the region surrounding the tryptophanyl residue of glycated, deffated, and glycated in the presence of fatty acids human serum albumin [33,36]. In order to study the REES effect, fluorescence spectra of glycated human serum albumin in the absence (af)gHSA and in the presence of fatty acids (af)gHSA_phys_, (af)gHSA_4S_, (af)gHSA_8S_, (af)gHSA_4US_, and (af)gHSA_8US_ excited at λ_ex_ = 290 nm, λ_ex_ = 295 nm, and λ_ex_ = 300 nm wavelengths were recorded (data not shown). 

Emission fluorescence spectra of (af)gHSA Trp-214 residue are different than for Trp-214 of (af)gHSA_phys_, (af)gHSA_4S_, (af)gHSA_8S_, (af)gHSA_4US_, and (af)gHSA_8US_ at all excitation wavelengths. A slight red-shift maximum emission fluorescence of (af)gHSA_phys_ (Δλ_em_ = 2 nm), (af)gHSA_4S_ (Δλ_em_ = 3 nm), (af)gHSA_8S_ (Δλ_em_ = 3 nm), (af)gHSA_4US_ (Δλ_em_ = 3 nm), and (af)gHSA_8US_ (Δλ_em_ = 3 nm) relative to (af)gHSA (Δλ_em_ = 6 nm) has been obtained. Higher shift for deffated, glycated albumin indicates that fatty acids present in the structure of albumin decreases the mobility of Trp-214 inducing changes of the albumin conformation. 

Similarly, a larger REES in the case of modified-glycated (gHSA_FRC_, Δλ_em_ = 5 nm) vs. non-modified (HSA, Δλ_em_ = 2 nm) human serum albumin was also observed in our previous study [28]. This suggest structural modifications in the hydrophobic pocket containing the tryptophanyl residue due to the glycation process, which contribute to stiffening of the Trp-214 environment or/and limited access to the polar solvent.

## 3. Materials and Methods

### 3.1. Reagents

Fatty-acid-free human serum albumin ((af)HSA, Lot No. 6312A) was purchased from MP Biomedicals LLC (Inc. Illkirch, France). Myristic acid (MYR, Lot No. R28576), oleic acid (OA, Lot No. 1912J), linoleic acid (LA, Lot No. 2353J), palmitic acid (PA, Lot No. 6798H), and stearic acid (SA, Lot No. 7729H) were provided by MP Biomedicals^TM^ (OH, USA). Sodium azide (NaN_3_, Lot No. BCBD6941V) and losartan (LOS, Lot No. SLBF5611V) were obtained from Sigma-Aldrich Chemical Co. (Darmstadt, Germany) and Sigma-Aldrich Chemical Co. (Shanghai, China), respectively. 

D(+)-glucose (GLC, Lot No. A0299881) was supplied by POCH S.A. (Gliwice, Poland). All chemicals were of the highest analytical quality. The stock solutions of MYR, OA, LA, PA, and SA were prepared by dissolving appropriate amounts in methanol for spectroscopy from Merck KGaA (Darmstadt, Germany, Lot No. 32373611/18).

### 3.2. In Vitro Glycation of Defatted Human Serum Albumin

Prior to in vitro glycation of defatted human serum ((af)HSA) albumin, all glass materials and spatulas were sterilized using a lab dryer at 100 °C prevent bacterial growth. Defatted human serum albumin ((af)HSA) in the presence of glucose (GLC) at 1.0 × 10^−5^ mol∙L^−1^ and 0.05 mol∙L^−1^ concentrations, respectively, were prepared in phosphate buffer solution (pH = 7.4, 0.05 mol∙L^−1^) in double-distilled water and in the presence of sodium azide (NaN_3_) (0.015 mol∙L^−1^). 

Solutions of protein were passed through a sterile Millex-GP syringe filters with 0.2 μm pores and then incubated in sterile closed tubes for a period of 21 days at aconstant temperature of 37 °C. After the incubation period, to remove excess unbound glucose, the solution of glycated human serum albumin (af)gHSA was dialyzed extensively against 0.05 mol∙L^−1^ phosphate buffer at pH = 7.4 for 24 h. The pH 7.4 ± 0.1 of buffer solution was confirmed by pH meter (FEP20 Metler Toledo). The absorbance of (af)HSA and (af)gHSA at 255 and 280 nm ratio was less than 0.5, indicating the purity of albumins.

### 3.3. Procedure of Preparation Fatty Acids Solutions

Stock solutions were made for fatty acids (FA) (oleic acid (OA), palmitic acid (PA), stearic acid (SA), myristic acid (MYR), and linoleic acid (LA)) with a concentration of 1 × 10^−3^ mol∙L^−1^ by dissolving the right amount of acid in methanol. In order to study the influence of fatty acids on the binding properties of glycated human serum albumin, five FA mixtures with differing contents were prepared in the phosphate buffer; saturated (FA_S_) and unsaturated (FA_US_) fatty acids (mix 1–5):

1. Fatty acids physiological mixture at 2.0 × 10^−5^ mol∙L^−1^ concentration containing FA mixtures at 6 × 10^−6^ mol∙L^−1^ PA, 0.5 × 10^−6^ mol∙L^−1^ SA, 0.5 × 10^−6^ mol∙L^−1^ MYR, 8 × 10^−6^ mol∙L^−1^ OA, and 5 × 10^−6^ mol∙L^−1^ LA concentrations—mix 1.
2. Fatty acids compound with a concentration of 4.1 × 10^−5^ mol∙L^−1^ with four times the amount of FA^S^ compared to the physiological mixture containing FA mixtures at 2.4 × 10^−5^ mol∙L^−1^ PA, 2.0 × 10^−6^ mol∙L^−1^ SA, 2.0 × 10^−6^ mol∙L^−1^ MYR, 8.0 × 10^−6^ mol∙L^−1^ OA, and 5.0 × 10^−6^ mol∙L^−1^ LA concentrations—mix 2.
3. A compound of fatty acids with a concentration of 6.9 × 10^−5^ mol∙L^−1^ with eight times the amount of FA_S_ compared to the physiological mixture containing 4.8 × 10^−5^ mol∙L^−1^ PA, 4.0 × 10^−6^ mol∙L^−1^ SA, 4.0 × 10^−6^ mol∙L^−1^ MYR, and 8.0 × 10^−6^ mol∙L^−1^ OA and with the concentration of 5.0 × 10^−6^ mol∙L^−1^ LA concentrations—mix 3.
4. A compound of fatty acids with the concentration of 5.9 × 10^−5^ mol∙L^−1^ with four times the amount of FA_US_ compared to the physiological mixture containing: 6.0 × 10^−6^ mol∙L^−1^ PA, 5.0 × 10^−5^ mol∙L^−1^ SA, 5.0 × 10^−5^ mol∙L^−1^ MYR, 3.2 × 10^−5^ mol∙L^−1^ OA, and 2.0 × 10^−5^ mol∙L^−1^ LA—mix 4.
5. A compound of fatty acids with the concentration of 1.11 × 10^−4^ mol∙L^−1^ with eight times the amount of FA_US_ compared to the physiological mixture containing 6.0 × 10^−6^ mol∙L^−1^ PA, 5.0 × 10^−5^ mol∙L^−1^ SA, 5.0 × 10^−5^ mol∙L^−1^ MYR, 6.4 × 10^−5^ mol∙L^−1^ OA, and 4.0 × 10^−5^ mol∙L^−1^ LA concentrations—mix 5.

### 3.4. Preparation of Samples for Fluorescence and UV-Vis Studies

Six glycated albumins (af)gHSA samples at 5 × 10^−6^ mol∙L^−1^ concentrations were prepared in the absence and in the presence of a suitable fatty acids mixture (albumin (af)gHSA_phys_: (af)gHSA + mix 1, (af)gHSA_4S_: (af)gHSA + mix 2, (af)gHSA_8S_: (af)gHSA + mix 3, (af)gHSA_4US_: (af)gHSA + mix 4, and (af)gHSA_8US_): (af)gHSA + mix 5). The content of methanol in the samples did not exceed 1% of the tested protein solution total volume.

The analysis of the interaction of losartan (LOS) with (af)gHSA, (af)gHSA_phys_, (af)gHSA_4S_, (af)gHSA_8S_, (af)gHSA_4US_, and (af)gHSA_8US_ was performed using the albumin fluorescence quenching method. Samples for fluorescence and absorbance measurements were made using the titration method. By the use of Hamilton syringe, a suitable volumes of LOS (3 μL in 10 portions) was added to 3 mL of albumins immediately before the fluorescence measurement. Due to the equipment limitations, the final molar ratio LOS:(af)gHSA, LOS:(af)gHSA_phys_, LOS:(af)gHSA_4S_, LOS:(af)gHSA_8S_, LOS:(af)gHSA_4US_, and LOS:(af)gHSA_8US_ was 10:1. The stock solution of losartan (LOS) at a 5 × 10^−3^ mol∙L^−1^ concentration was prepared in distilled water.

### 3.5. Instruments and Measurements Conditions

The fluorescence and absorbance measurements of the samples were recorded at 37 °C using a JASCO spectrofluorimeter FP-6500 equipped with a Peltier thermostat (∆t ± 0.2 °C) (error apparatus ± 1.5 nm) and JASCO spectrophotometer V-760 (the correction of the error of the apparatus for the wavelength and photometric readings was equal to ±0.3 nm and ±0.002 Abs. at 0.5 Abs), respectively, and standard quartz cuvettes. The fluorescence spectra presented in the paper were corrected for the phosphate buffer using the Spectra Manager program and then analyzed using OriginPro version 8.5 SR1 software (Northampton, MA, USA). The results of the study were expressed as a mean ± relative standard deviation (RSD) from three independent experiments.

The emission fluorescence spectra of tryptophanyl (Trp-214) and thyrosyl (Tyr) residues of the proteins ((af)gHSA, (af)gHSA_phys_, (af)gHSA_4S_, (af)gHSA_8S_, (af)gHSA_4US_, and (af)gHSA_8US_) were recorded at the excitation wavelength λ_ex_ = 275 nm (λ_em_ = 285–400 nm), and the fluorescence spectra of the Trp-214 were measured at λ_ex_ = 295 nm (λ_em_ = 305–400 nm). The synchronous fluorescence spectra were obtained considering the wavelength intervals ∆λ = 15 nm and ∆λ = 60 nm to evidence the protein fluorophores Tyr residues and Trp-214 residue, respectively (∆λ—difference between emission (λ_em_) and excitation (λ_ex_) wavelength). 

The measurements were conducted when the spectral width of the band (for monochromator of excitation and emission radiation) was equal to 3 nm with a sample scanning speed of 100 nm/min. The Red Edge Excitation Shift (REES) of (af)gHSA was compared to (af)gHSA_phys_, (af)gHSA_4S_, (af)gHSA_8S_, (af)gHSA_4US_, and (af)gHSA_8US_) with the use of λ_ex_ = 290 nm, λ_ex_ = 295 nm, and λ_ex_ = 300 nm. The measurement ranges of all emission spectra were recorded from 310 to 400 nm and the slit widths were 3 nm/3 nm.

The degree of macromolecule fluorescence quenching by the LOS was determined relative to the fluorescence of the non-ligand albumin solutions. Due to the inner filter effect (IFE) caused by the presence of the drug, the recorded fluorescence was corrected using the following formula (Equation (1)) [19]:

(1) $$F_{\mathrm{cor}}=F_{\mathrm{obs}}\cdot 10^{\left(\frac{A_{\mathrm{ex}}+A_{\mathrm{em}}}{2}\right)}$$

where $F_{\mathrm{cor}}$ and $F_{\mathrm{obs}}$ are the corrected and observed fluorescence intensity, respectively; $A_{\mathrm{ex}}$ and $A_{\mathrm{em}}$ are the absorbance at excitation (λ_ex_ = 275 nm or λ_ex_ = 295 nm) and emission wavelength for (af)gHSA, (af)gHSA_phys_, (af)gHSA_4S_, (af)gHSA_8S_, (af)gHSA_4US_, and (af)gHSA_8US_, respectively.

### 3.6. Analysis of Fluorescence Spectra—Calculation of the Stern–Volmer and Association Constants

Based on the calculated fluorescence emission intensities in the absence and in the presence of fatty acids glycated human serum albumin, curves of (af)gHSA, (af)gHSA_phys_, (af)gHSA_4S_, (af)gHSA_8S_, (af)gHSA_4US_, and (af)gHSA_8US_ fluorescence quenching by losartan (LOS) ($\frac{F}{F_{0}}\;$ vs. ligand:albumin molar ratio, where F and $F_{0}$ is the fluorescence intensity at the maximum wavelength of albumin in the presence and absence of a quencher, respectively) were drawn.

The quenching effect (static and/or dynamic) fluorescence of (af)gHSA, (af)gHSA_phys_, (af)gHSA_4S_, (af)gHSA_8S_, (af)gHSA_4US_, and (af)gHSA_8US_, the Stern–Volmer constants K_SV_, the bimolecular quenching rate constants k_q_ (k_q_ = K_SV_/τ_0_), and maximum available fluorescence fraction of all albumin $f_{a}$ fluorophores were analyzed on the basis of the Stern–Volmer equation (Equation (2)) [20]:

(2) $$\frac{F_{0}}{F}=1+K_{\mathrm{SV}}\cdot \left[L\right]=1+k_{q}\tau_{0}\cdot \left[L\right]$$

where $k_{q}$ is the bimolecular quenching rate constant [mol^−1^∙L∙s^−1^]; $\tau_{0}$ is the average fluorescence lifetime of albumin without of quencher $\tau_{0}$ = 6.0 × 10^−9^ s [22]; $K_{\mathrm{SV}}$ is the Stern–Volmer constant [mol^−1^∙L]; and [L] is the ligand concentration [mol∙L^−1^] where $\left[L\right]=\left[L_{b}\right]+[L_{f}]$, $\left[L_{b}\right]$ and $\left[L_{f}\right]$ are the bound and free (unbound) drug concentrations [mol∙L^−1^].

The quenching parameters (K_SV_, k_q_) and $f_{a}$ for a system with non-linear Stern–Volmer relationship were calculated using the Stern–Volmer equation modified by Lehrer (Equation (3)) [21]:

(3) $$\frac{F_{0}}{∆F}=\frac{1}{\left[L\right]}\cdot \frac{1}{f_{a}}\cdot \frac{1}{K_{\mathrm{SV}}}+\frac{1}{f_{a}}$$

where $f_{a}$ is the fractional maximum protein fluorescence accessible for the quencher.

Isotherms of losartan binding to glycated human serum albumin in the absence and in the presence of fatty acids were obtained based on the graph of the function $r=f\left(\left[L_{f}\right]\right)$, where $r=\frac{\left[L_{b}\right]}{\left[\left(\mathrm{af}\right)\mathrm{gHSA}\right]}$ is the number of ligands moles bound per mole of protein molecule; $\left[L_{b}\right]=\frac{∆F}{∆F_{\max}}\times \left(\mathrm{af}\right){\mathrm{gHSA}}_{\mathrm{total}},\;∆F$ is the difference between $F_{0}$ and F; ${∆F}_{\max}$ (the maximal fluorescence change with complete saturation) is evaluated from the linear part of the $\frac{1}{∆F}$ vs. $\frac{1}{\left[L\right]}$; and [(af)gHSA] is serum albumin concentration [mol∙L^−1^] [29].

From the Scatchard (Equation (4)) [37] and the Klotz (Equation (5)) [38] curves, the values of K_a_ association constants and n the number of binding sites in the albumin molecule were determined.

(4) $$\frac{r}{\left[L_{f}\right]}=n\cdot K_{a}-K_{a}\cdot r$$

(5) $$\frac{1}{r}=\frac{1}{n}+\frac{1}{n\cdot K_{a}\cdot [L_{f}]}$$

Hill’s coefficient $n_{H}$ was determined on the basis of the Hill method (Equation (6)) [39]:

(6) $$\log\left(\frac{r}{1-r}\right)=n_{H}\cdot \log[L_{f}]+{\mathrm{logK}}_{a}$$

## 4. Conclusions

Fluorescence spectroscopic measurements confirmed that losartan (LOS) interacts with glycated human serum albumin (af)gHSA, both in the absence and in the presence of fatty acids ((af)gHSA_phys_, (af)gHSA_4S_, (af)gHSA_8S_, (af)gHSA_4US_, and (af)gHSA_8US_) and quenches albumin fluorescence intensity via a static quenching mechanism. Based on the obtained data, we can conclude that LOS molecules locate closer to the fluorophores of glycated fatted by fatty acids physiological mixture albumin (af)gHSA_phys_ compared with glycated defatted albumin (af)gHSA fluorophores. 

They also locate at different distances to fluorophores of glycated albumin containing various amounts of saturated and unsaturated fatty acids. Moreover, LOS binds not only to its specific binding sites in albumins but also non-specifically interacts with the hydrophobic fragments of its surface and has the same affinity for (af)gHSA and (af)gHSA_phys_ albumin binding sites. A higher concentration of both saturated and unsaturated fatty acids in glycated albumin reduces the stability of the complex formed with the drug. 

According to our results, we can also conclude that, in the case of drugs with a high degree of protein binding (such as losartan) or with a narrow therapeutic index, no changes in the concentration of the albumin in the blood result in a change in the drug’s free fraction concentration responsible for therapeutic effects. The presented study allowed for determination of the binding capacity of albumin that is structurally changed by glycation and/or in the presence of fatty acids. This can be used to model drug–protein interactions simulating pathological conditions in the body.

## Figures and Tables

**Table 1:** Fluorescence quenching of LOS-(af)gHSA, LOS-(af)gHSA_phys_, LOS-(af)gHSA_4S_, LOS-(af)gHSA_4US_, LOS-(af)gHSA_8S_, an LOS-(af)gHSA_8US_ systems and the fluorescence quenching percentage at λ_ex_ = 275 nm and λ_ex_ = 295 nm excitation wavelength; albumin and losartan concentrations were 5 × 10^−6^ mol∙L^−1^ and 5 × 10^−5^ mol∙L^−1^, respectively.

Ligand–Albumin System	λ_ex_ = 275 nm	λ_ex_ = 295 nm	Difference between λ_ex_ = 275 nm and λ_ex_ = 295 nm
	Fluorescence Quenching Percentage (%)		
LOS-(af)gHSA	48.36%	55.99%	7.63%
LOS-(af)gHSA_phys_	59.34%	77.80%	18.46%
LOS-(af)gHSA_4S_	55.89%	67.93%	12.04%
LOS-(af)gHSA_8S_	49.08%	52.66%	3.58%
LOS-(af)gHSA_4US_	45.91%	49.63%	3.72%
LOS-(af)gHSA_8US_	34.20%	26.23%	7.97%

**Table 2:** Stern–Volmer constants K_SV_ (mol^−1^∙L), bimolecular quenching rate constants k_q_ (mol^−1^∙L∙s^−1^) and maximum available fluorescence fraction f_a_ of all albumin fluorophores calculated for the LOS-(af)gHSA, LOS-(af)gHSA_phys_, LOS-(af)gHSA_4S_, LOS-(af)gHSA_4US_, LOS-(af)gHSA_8S_, and LOS-(af)gHSA_8US_ systems; λ_ex_ = 275 nm and λ_ex_ = 295 nm.

**λ_ex_ = 275 nm**	K_SV_ ± RSD *^)^ × 10^4^ **(mol^−1^∙L)**	^a^ k_q_ ± RSD *^)^ × 10^12^ **(mol^−1^∙L∙s^−1^)**	f_a_ ± RSD *^)^
LOS-(af)gHSA	1.87 ± 0.05	3.12 ± 0.08	0.96 ± 0.02
LOS-(af)gHSA_phys_	2.95 ± 0.03	4.92 ± 0.06	0.99 ± 0.01
LOS-(af)gHSA_4S_	2.54 ± 0.01	4.23 ± 0.01	1.01 ± 0.01
LOS-(af)gHSA_8S_	1.95 ± 0.02	3.25 ± 0.03	1.02 ± 0.01
LOS-(af)gHSA_4US_	1.68 ± 0.02	2.80 ± 0.03	1.01 ± 0.01
LOS-(af)gHSA_8US_	1.04 ± 0.02	1.73 ± 0.03	1.01 ± 0.01
**λ_ex_ = 295 nm**	K_SV_ ± RSD *^)^ × 10^4^ **(mol^−1^∙L)**	^a^ k_q_ ± RSD *^)^ ** × 10^12^** **(mol^−1^∙L∙s^−1^)**	f_a_ ± RSD *^)^
LOS-(af)gHSA	2.57 ± 0.02	4.28 ± 0.03	1.01 ± 0.01
LOS-(af)gHSA_phys_	2.65 ± 0.01	4.42 ± 0.01	1.39 ± 0.01
LOS-(af)gHSA_4S_	2.08 ± 0.02	3.47 ± 0.03	1.34 ± 0.02
LOS-(af)gHSA_8S_	2.22 ± 0.06	3.70 ± 0.09	1.04 ± 0.01
LOS-(af)gHSA_4US_	1.94 ± 0.05	3.23 ± 0.08	1.03 ± 0.01
LOS-(af)gHSA_8US_	0.71 ± 0.01	1.18 ± 0.01	1.00 ± 0.01

*^)^ relative standard deviation; **^a^** calculated using: $k_{q}=\frac{K_{\mathrm{SV}}}{\tau_{0}}$, where $\tau_{0}$ = 6.0 × 10^−9^ s [22]—the average fluorescence lifetime of albumin without quencher.

**Table 3:** Association constants K_a_ (mol^−1^∙L), mean number of LOS moles bound with one mole of (af)gHSA, (af)gHSA_phys_, (af)gHSA_4S_, (af)gHSA_4US_, (af)gHSA_8S_, and (af)gHSA_8US_ (*n*), the Hill coefficient (n_H_) in The LOS–albumin systems; λ_ex_ = 275 nm, λ_ex_ = 295 nm.

	Scatchard Method		Klotz Method		Hill Method
λ_ex_ = 275 nm	K_a_ ± RSD *^)^ × 10^4^ (mol^−1^∙L)	*n* ± RSD *^)^	K_a_ ± RSD *^)^ × 10^4^ (mol^−1^∙L)	*n* ± RSD *^)^	n_H_ ± RSD *^)^
LOS-(af)gHSA	3.75 ± 0.02	0.93 ± 0.01	3.74 ± 0.01	0.94 ± 0.01	0.96 ± 0.01
LOS-(af)gHSA_phys_	3.66 ± 0.03	0.93 ± 0.01	3.64 ± 0.01	0.93 ± 0.01	0.96 ± 0.01
LOS-(af)gHSA_4S_	2.61 ± 0.02	0.94 ± 0.01	2.59 ± 0.01	0.94 ± 0.01	0.97 ± 0.01
LOS-(af)gHSA_8S_	1.52 ± 0.03	0.95 ± 0.02	1.51 ± 0.02	0.95 ± 0.01	0.99 ± 0.01
LOS-(af)gHSA_4US_	1.58 ± 0.04	0.92 ± 0.03	1.49 ± 0.02	0.96 ± 0.02	0.98 ± 0.01
LOS-(af)gHSA_8US_	0.53 ± 0.02	0.94 ± 0.04	0.50 ± 0.02	0.99 ± 0.04	0.99 ± 0.01
**λ_ex_ = 295 nm**	K_a_ ± RSD *^)^ **× 10^4^ (mol^−1^∙L)**	*n* ± RSD *^)^	K_a_ ± RSD *^)^ **× 10^4^ (mol^−1^∙L)**	*n* ± RSD *^)^	n_H_ ± RSD *^)^
LOS-(af)gHSA	2.82 ± 0.03	0.95 ± 0.02	2.87 ± 0.01	0.94 ± 0.01	0.98 ± 0.01
LOS-(af)gHSA_phys_	3.25 ± 0.02	0.93 ± 0.01	3.22 ± 0.01	0.94 ± 0.01	0.96 ± 0.01
LOS-(af)gHSA_4S_	2.41 ± 0.04	0.95 ± 0.02	2.44 ± 0.03	0.94 ± 0.02	0.98 ± 0.01
LOS-(af)gHSA_8S_	1.38 ± 0.04	0.95 ± 0.04	1.36 ± 0.03	0.96 ± 0.03	0.99 ± 0.01
LOS-(af)gHSA_4US_	1.31 ± 0.02	0.93 ± 0.02	1.26 ± 0.02	0.96 ± 0.01	0.98 ± 0.01
LOS-(af)gHSA_8US_	0.62 ± 0.02	1.04 ± 0.05	0.67 ± 0.02	0.98 ± 0.03	1.00 ± 0.01

*^)^ relative standard deviation.

**Table 4:** Intensity of 5 × 10^−6^ mol∙L^−1^ (af)gHSA, (af)gHSA_phys_, (af)gHSA_4S_, (af)gHSA_4US_, (af)gHSA_8S_, and (af)gHSA_8US_ emission (λ_ex_ = 275 nm, λ_ex_ = 295 nm) and synchronous (Δλ = 15 nm and Δλ = 60 nm) fluorescence spectra.

5 × 10^−6^ mol∙L^−1^	λ_ex_ = 275 nm		λ_ex_ = 295 nm		Δλ = 15 nm		Δλ = 60 nm	
	λ_max_ (nm)	F_max_	λ_max_ (nm)	F_max_	λ_max_ (nm)	F_max_	λ_max_ (nm)	F_max_
(af)gHSA	321	78.83	335	22.86	299	45.62	337	71.22
(af)gHSA_phys_	320	89.78	332	27.72	300	51.75	337	78.40
(af)gHSA_4S_	321	90.73	331	27.93	300	51.88	337	78.91
(af)gHSA_8S_	319	102.64	326	31.85	300	57.90	336	82.85
(af)gHSA_4US_	319	104.57	327	33.12	300	56.68	336	82.90
(af)gHSA_8US_	319	107.61	326	34.27	300	57.39	336	83.84

## Data Availability

Not applicable.

## Sample Availability

Samples of the compounds are available from the authors.
